# A Reliable Wireless Control System for Tomato Hydroponics

**DOI:** 10.3390/s16050644

**Published:** 2016-05-05

**Authors:** Hirofumi Ibayashi, Yukimasa Kaneda, Jungo Imahara, Naoki Oishi, Masahiro Kuroda, Hiroshi Mineno

**Affiliations:** 1 Graduate School of Informatics, Shizuoka University, 3-5-1 Johoku, Naka-ku, Hamamatsu, Shizuoka 432-8011, Japan; 2 Graduate School of Integrated Science and Technology, Shizuoka University, 3-5-1 Johoku, Naka-ku, Hamamatsu, Shizuoka 432-8011, Japan; kaneda@minelab.jp; 3 Shizuoka Prefectural Research Institute of Agriculture and Forestry, 678-1 Tomioka, Iwata, Shizuoka 438-0803, Japan; jungo1_imahara@pref.shizuoka.lg.jp (J.I.); naoki1.ohishi@gmail.com (N.O.); 4 National Institute of Information and Communications Technology, 4-2-1 Nukui-Kitamachi, Koganei 184-8795, Japan; marsh@nict.go.jp; 5 JST, PRESTO, 4-1-8 Honcho, Kawaguchi, Saitama 332-0012, Japan

**Keywords:** sensor network, actuator control, highly reliable wireless communication, agricultural system, 400 MHz band

## Abstract

Agricultural systems using advanced information and communication (ICT) technology can produce high-quality crops in a stable environment while decreasing the need for manual labor. The system collects a wide variety of environmental data and provides the precise cultivation control needed to produce high value-added crops; however, there are the problems of packet transmission errors in wireless sensor networks or system failure due to having the equipment in a hot and humid environment. In this paper, we propose a reliable wireless control system for hydroponic tomato cultivation using the 400 MHz wireless band and the IEEE 802.15.6 standard. The 400 MHz band, which is lower than the 2.4 GHz band, has good obstacle diffraction, and zero-data-loss communication is realized using the guaranteed time-slot method supported by the IEEE 802.15.6 standard. In addition, this system has fault tolerance and a self-healing function to recover from faults such as packet transmission failures due to deterioration of the wireless communication quality. In our basic experiments, the 400 MHz band wireless communication was not affected by the plants’ growth, and the packet error rate was less than that of the 2.4 GHz band. In summary, we achieved a real-time hydroponic liquid supply control with no data loss by applying a 400 MHz band WSN to hydroponic tomato cultivation.

## 1. Introduction

Nowadays, numerous kinds of sensors are being developed for many different applications, and many sensor-based control systems for agriculture have been implemented taking advantage of advances in information and communication technology (ICT) [1,2,3,4]. These control systems work automatically to control the growth of the plants instead of requiring the constant attention of farmers. Recent control systems aim to produce high-quality vegetables and fruits by precise control. In Japan, there is already a greenhouse environmental control system [5] using an ubiquitous environmental control system (UECS) [6]. This system can collect a variety of environmental data by adding data from any sensor conforming to UECS standards. However, wired communications is the mainstream form of communication between sensors and sink node when communicating using UECS standards. Thus, there is the problem of the high cost burden on farmers due to the installation costs for items such as wiring when installing a large number of sensors, although the communication reliability is guaranteed.

Meanwhile, research on sensing the agricultural environments using wireless sensor networks (WSNs) [7,8,9,10] is also being conducted. For example, there are some studies about sensing soil moisture using a WSN [11] or irrigation control by sensing soil moisture and temperature, and air temperature [12]. In particular, when installing a WSN in a horticulture environment, packet losses may occur due to a decrease in receiving power caused not only by obstacles such as metal pipes and the plants themselves, but also the installation position or characteristics of the radio antenna [13]. Moreover, the 2.4 GHz band has the property of being easily absorbed in water [14]. A reliable environment monitoring system using a WSN aiming for at least 99.99% system availability has also been promoted [15], but it is necessary to determine the reliability of the actuator control as well as the sensing.

WSNs for agricultural applications generally use ZigBee [16], which is based on the layers specified in the IEEE 802.15.4 wireless communication standard. This is because ZigBee has low power consumption and is easy to operate. A study on improving the packet arrival rate by introducing time-slotted or frequency division multiplexing [17] has been carried out, and its ideas have been combined with studies on packet collision avoidance or the traffic distribution mechanism [18]. However, these studies mostly evaluate the results by simulation, and thus they do not take into account the real environment where the wireless communication environment changes depending on the amount of vegetation, climate, and installed products.

In recent years, there has been a focus on the use of lower frequency bands. By lowering the frequency, the wavelength becomes longer, and the diffraction by obstacles is better. There is research on potato field sensing using the 433 MHz band, where the radio propagation was good, even in high humidity environments [19]. In Japan, there is the 429 MHz band, which is used for medical telemetry, and there is the IEEE802.15.6 standard for using this frequency band [20]. The IEEE 802.15.6 standard has reserved transmission slot (using time division multiple access or TDMA) and retransmission processing functions. Therefore, it may be possible to build a highly reliable WSN using the 400 MHz band and IEEE 802.15.6 standard, even when there are many obstacles.

In this paper, we propose a highly reliable wireless control system for hydroponic tomato cultivation. We implemented a system prototype that can be reliably controlled using the 400 MHz band and IEEE 802.15.6 standard. In addition, this system has high fault tolerance and self-healing capability in response to faults. We operated the implemented system in a greenhouse environment and confirmed whether the system can run without actuator control failure during the cultivation period. Finally, we considered whether the wireless communication is affected by the plants.

## 2. Overview of the Tomato Hydroponics Control System

### 2.1. Overview of the Tomato Hydroponics Control System

The tomato hydroponics control system is a system prototype of a highly reliable wireless environmental control system. Hydroponics is a kind of plant cultivation method without soil. Hydroponic cultivation usually produces yield increases [21], but it needs precise control to produce high quality crops. For example, there is a possibility that the size of the crop will be small or the crop will die if the system supplies too little hydroponic liquid. On the other hand, if the system supplies too much, it may produce unusually shaped crops. Therefore, reliable system operation and hydroponic supply control is required.

**Figure 1:**
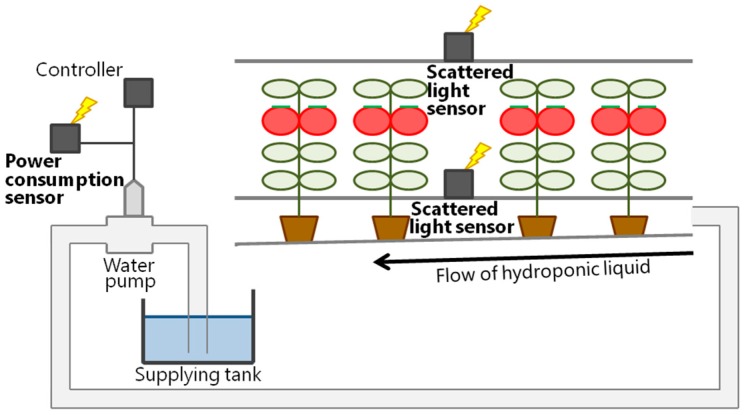
Sensor layout of the tomato hydroponics control system.

An overview of our system is shown in Figure 1. Our tomato hydroponics control system estimates the condition of the tomato plants in real-time using sensors, and the system then supplies the appropriate amount of hydroponic liquid for the tomatoes. The amount of hydroponic liquid supplied is determined by the evapotranspiration, which is calculated from the vapor pressure deficit (VPD) and relative light intensity (RLI). Evapotranspiration means the loss of water by plant leaves. The VPD is calculated from the temperature and relative humidity. RLI is the ratio of the amount of scattered light at the top and bottom of the tomato communities. By installing scattered light sensor nodes at the top and bottom of the tomatoes, the amount of transpiration can be estimated with an error of less than 3% from the amount of photosynthesis and transpiration using the data collected by the scattered light sensors [22]. 

### 2.2. System Requirement Analysis

**Figure 2:**
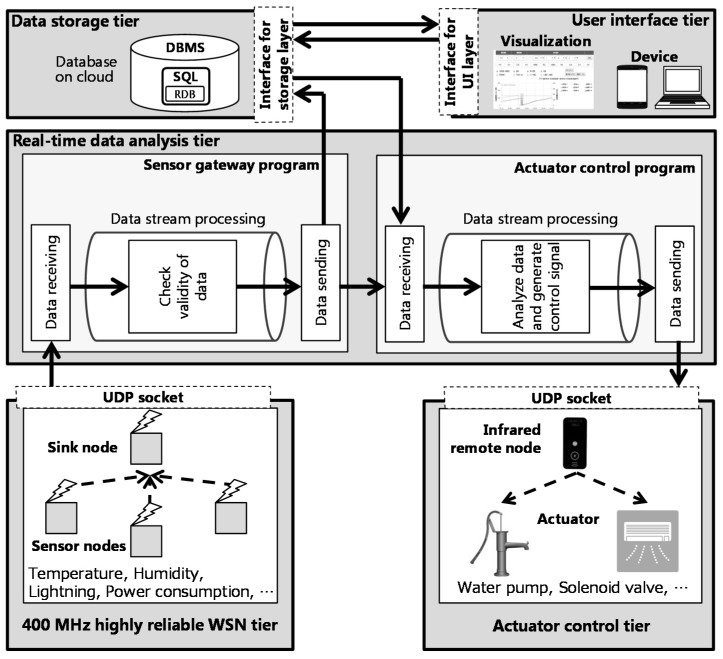
System architecture.

Our proposed system is an environmental control system for the growth control of tomatoes. The architecture of our proposed system is shown in Figure 2. Sensor nodes collect greenhouse environment data on the state of the crops. Then, these data are sent to the sensor gateway through the sink node that aggregates the data from each sensor node. The sensor gateway determines whether the sensor data is correct, and then it sends the sensor data to the storage in the cloud and the actuator control program. Finally, the system controls the actuators that feed the hydroponic liquid according to the control information. We use wireless sensor nodes to facilitate the installation of the system and reduce wiring costs. The control information is input by the user who receives the environmental data. Our system allows the user to view the environmental data and input control information from anywhere through a Web browser.

Many growth control systems are controlled by times that are set in advance. However, the production of high-quality crops requires a control system based on real-time environmental information because the state of the greenhouse environment and the growth of plants change with time. For example, evapotranspiration per minute under sunny daytime conditions is several milliliters, so the system has to be able to irrigate plants based on real-time evapotranspiration of the plants every minute. To produce high quality tomatoes, it is necessary to manage the hydroponic liquid reliably and delicately. If the system stops and cannot control the hydroponic liquid supply, the tomato harvest and quality are reduced. As the proposed system is a feedback control system using sensor data, if sensor data or control data loss occurs, the system cannot control the nutrient supply, so it is necessary to reduce packet losses as much as possible.

In crop control systems it is necessary to operate the system continuously even if the user cannot always monitor the system operation status such as when operating at night. When the system fails and cannot control the actuator normally, the quality of crops may decrease. Therefore, it is necessary to operate the system continuously during the cultivation period.

The greenhouse environment is hot and humid; the maximum temperature can be 40 °C or more, and the relative humidity is over 90%. Therefore, it is necessary for electronic equipment to have a waterproof layer and heat protection, even though it is not expected to be used outdoors. Furthermore, the system cannot control the actuator if the environmental data is lost because this system controls the actuator in real time by using the sensor data. Therefore, for reliable actuator control, it is necessary to suppress the data loss caused by not only wireless communication errors in the WSN, but also buffer overflow in the real-time data analysis tier due to an increase in the number of processed data per unit time.

On the other hand, it is assumed that the users set the control information for the actuators based on the collected environmental data. The users who can connect to the Internet can view the environmental data and change control information from any location. Therefore, users’ convenience may be improved by managing these environmental data and actuator control information in a cloud server. However, when the Internet fails, control signals do not reach the control program, and the system cannot control the actuators. Also, if the environmental data is only stored in the cloud server database management system (DBMS), data loss will occur when the system cannot connect to the Internet. Note that the cloud server is in our laboratory for easy maintenance at this time, and the server is accessed through the Internet by using the portable 3G/4G wireless router at the greenhouse located about 10 km away from our campus. Therefore, it is necessary that the data not be lost even if failure occurs in the uplink and downlink direction of the communication path and cloud servers to achieve reliable environmental control by backup storage in the local network. The required specifications for a reliable wireless environmental control system in this study are summarized below:

1. By using a large number of sensors to collect environmental data in the greenhouse environment, the collected environmental data is stored in the cloud server, and it is possible to browse the data from any location through a visualization interface.
2. The environmental control system in the field environment can be configured by system users via the control information setting interface from any location, and actuator control can be performed based on the control information set.
3. The system requires that a system failure hardly ever occurs, and it has a self-healing function in the event of failure to prevent the crop being affected by the system stop.

### 2.3. A 400 MHz Highly Reliable Wireless Sensor Network

**Figure 3:**
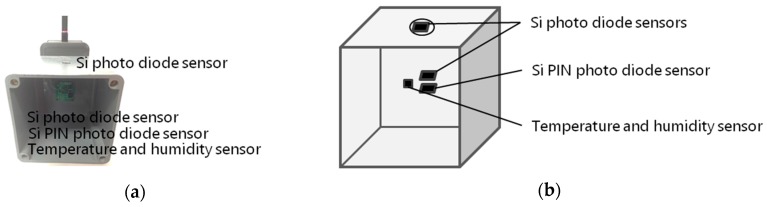
Scattered light sensor node. (**a**) photograph; (**b**) overview.

The 400 MHz highly reliable wireless sensor network is composed of some sensor nodes and a sink node. This satisfies requirements 1 and 3. In the tomato hydroponics control system, we used temperature, humidity, and light intensity sensors to estimate hydroponic liquid supply. The amount of hydroponic liquid supply is determined on the basis of the evapotranspiration, and the evapotranspiration is calculated from the VPD, which is determined by the temperature and relative humidity, and the RLI, which is determined by the scattered lighting. Therefore, we used scattered light sensor nodes, as shown in Figure 3a, to collect these environmental data. The scattered light sensor node has two Si photodiode sensors (S1133, Hamamatsu Photonics, Hamamatsu, Japan), a Si PIN photo diode sensor (S2506, Hamamatsu Photonics), and a temperature and humidity sensor (SHT21, Sensirion, Zurich, Switzerland) as shown in Figure 3b. One of the Si photodiode sensors is attached to the top of the box and measures the amount of outdoor solar radiation. The other Si photodiode sensor is attached to the inside of the box and measures the strength of light scattered in the air. Moreover, to measure a wider range of wavelength, we used a Si PIN photodiode sensor [23]. The Si photodiode sensors can measure wavelengths from 320 nm to 1000 nm, while the Si PIN photodiode sensor can measure wavelengths from 760 nm to 1100 nm.

**Figure 4:**
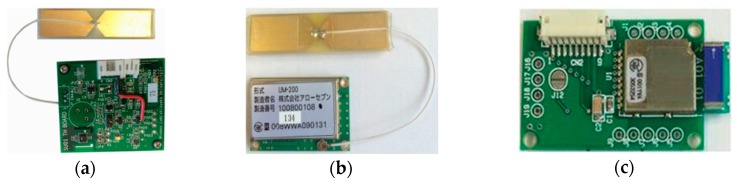
(**a**) Temperature and humidity sensor node, (**b**) 2.4 GHz band wireless module, and (**c**) 400 MHz band wireless module.

In addition, we prepared a temperature and humidity sensor node that can change the wireless module for evaluation. Although this sensor node is not used for control, it is used for comparison of the radio frequency and communication system. This sensor node is attached to a wireless module (Figure 4a) using the 2.4 GHz band and IEEE 802.15.4-2006 standard (Figure 4b) [24].

This wireless module is connected to the sensor node with a generic connector, and the communication system between the sensor node and the wireless module uses the UART protocol. Therefore, we developed a substrate that can be connected to the 400 MHz band wireless module (Figure 4c). The specifications of each wireless module are given in Table 1. If we could use 2.4 GHz wireless modules implemented based on the same standard as 400 MHz wireless modules, we could only evaluate the effect due to the difference in the frequency.

Since the proposed system is a feedback control system using sensor data, it is necessary to collect data without losses to ensure actuator control. Therefore, we introduced the 400 MHz band under the ARIB T67 radio standard [25] for highly reliable wireless communication. The 400 MHz band has high diffraction from obstacles because the wavelength of the 400 MHz band is longer than that of the 2.4 GHz band. Thus, it assumes that reliable wireless communication can be realized even if the sensor nodes are in the plants. On the other hand, there is the disadvantage that the bit rate is low because the bandwidth of the 400 MHz band is narrower than that of the 2.4 GHz band. Hence, it is not suitable for the transfer of large size data such as pictures and videos, but we think that it is not a big problem because we assumed the system would deal with only a few bytes of sensor data.

**Figure 5:**
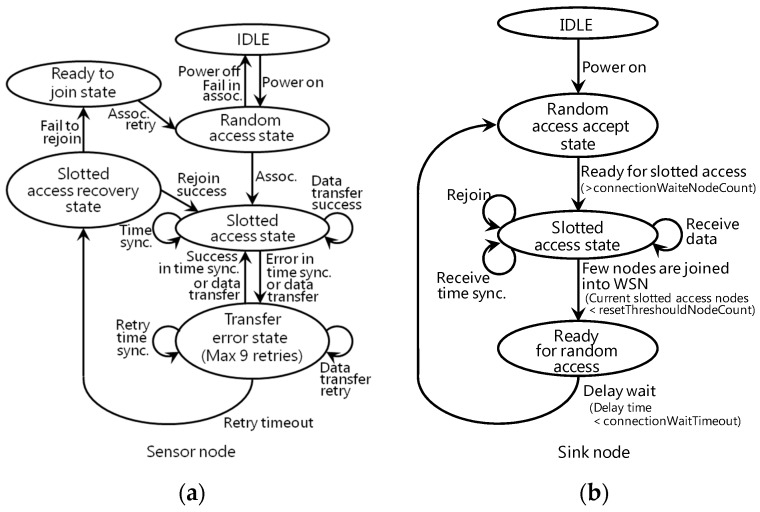
State diagram: (**a**) sensor node; (**b**) sink node.

Additionally, this wireless module is provided with two channel access mechanisms, random access with carrier sense multiple access/collision avoidance (CSMA/CA) and slotted access with TDMA, to accommodate many sensor nodes quickly and ensure reliable packet delivery. The state transition diagram of a sensor node is shown in Figure 5a, and a sink node is shown in Figure 5b [26]. First, the sink node is in the “Random access accept state” when it is powered on. When a sensor node is powered on and joined into a WSN, it uses CSMA/CA in the “Random access state”. If the channel is busy or a collision occurs, the node waits for a constant time. Then all sensor nodes are joined into the WSN, and each sensor node sends a packet with TDMA in the “Slotted access state”. As all sensor nodes send or receive the packets in assigned time slots, the data transmission is guaranteed to be collision-free in a star topology. Our system does not assume the situation in which each sensor node communicates with the others. Therefore, we used the star topology with TDMA for easy maintenance. Although there are wireless modules with time slotted channel hopping (TSCH) mesh networking technologies, it was very difficult to analyze the details of characteristics compared with our 400 MHz wireless modules.

If the packet does not reach the sink node or an ACK does not reach the sensor node, the sensor node transitions to the “Transfer error state”. In the “Transfer error state”, the sensor node resends the packet up to nine times in our prototype implementation. If retransmission is successful, the sensor node goes back to the “Slotted access state”. If the packet does not succeed after nine retransmissions, the sensor nodes transition to the “Random access state” via the “Slotted access recovery state” and “Ready to join state”. At the same time, the sink node does not receive the packet from a sensor node for a certain time; in other words, if after nine retransmissions the message does not reach the sink node, the sink node regards the sensor node as a breakaway node based on the configuration file. Then, if the number of the breakaway nodes exceeds the threshold of the configuration file, the sink node transitions to the “Random access accept state” via the “Ready for random access state”. Finally, when the sensor nodes and the sink node transition to the “Random access state” at the same time, the WSN is rebuilt. This failure recovery is performed automatically on the WSN side. For this reason, the system user does not need to recover the system.

### 2.4. Real-Time Data Analysis

The real-time data analysis tier includes two main programs: the sensor gateway program and the actuator control program. Input and output between each program are performed by Internet protocol suite or serial communication. Therefore, by increasing the number of modules independent of each program, it is possible to easily deal with sensor network changes and control algorithms.

**Figure 6:**
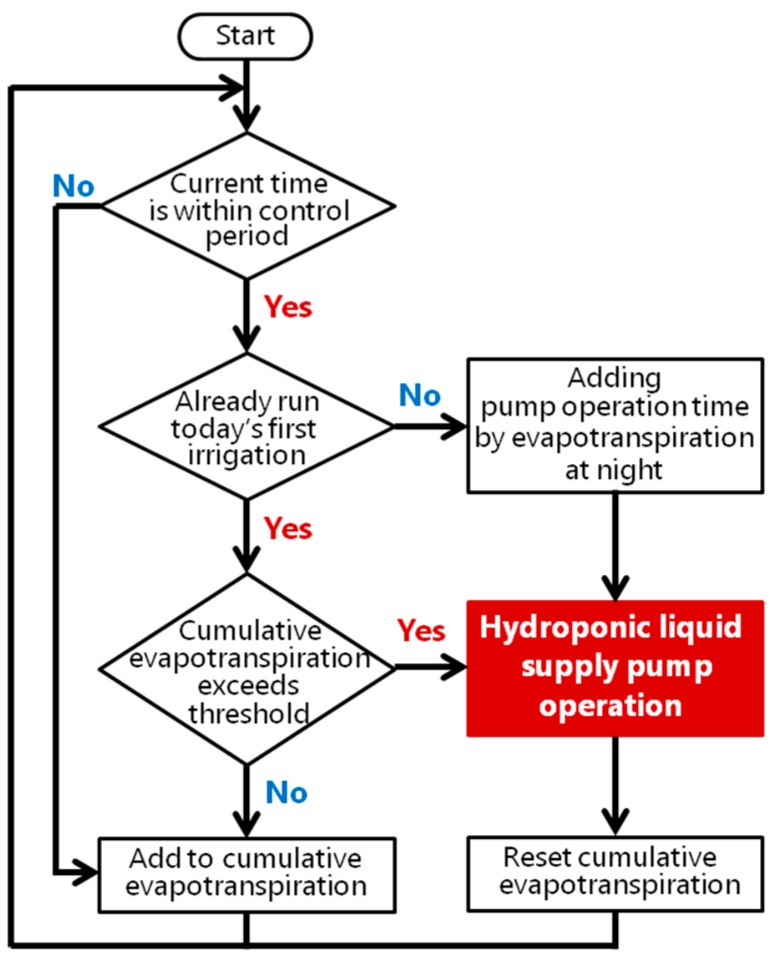
Algorithm for controlling the hydroponic liquid supply.

A sensor gateway verifies whether the packet from the sensor nodes has been broken and registers the sensor data in the storage on the cloud. It also sends the data to the actuator control program by user datagram protocol (UDP). The actuator control program analyzes the sensor data and estimates the current state of the plant. In this system, the actuator control program estimates the plants evapotranspiration per minute. If the accumulated evapotranspiration exceeds a threshold set by the users, the actuator control program sends a signal to the infra-red remote control to run the pump that supplies the hydroponic liquid. This algorithm is shown in Figure 6. This satisfies requirement 2.

A real-time data analysis tier is put in the greenhouse environment. This environment will be hot and humid to help the plants to grow. Therefore, we adopted OpenBlocks A7 (OBSA7P/J7, Plat’Home, Tokyo, Japan), guaranteed to operate up to 55 °C as a microserver to run both programs. Furthermore, the sensor gateway program and control decision program not only run with an executable format, but also minimize other unnecessary processes in system operation and reduce CPU and memory usage to suppress the occurrence of thermal runaways and reduce the risk of failure.

## 3. System Evaluation

### 3.1. Basic Experiment

**Figure 7:**
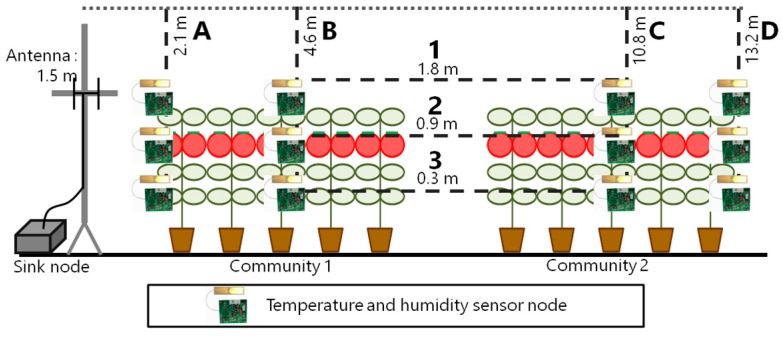
Sensor position for the basic experiment.

First, we performed a basic experiment to compare wireless modules. We placed temperature and humidity sensor nodes in a greenhouse at Shizuoka Prefectural Research Institute of Agriculture and Forestry (Iwata, Japan). The sensor nodes were placed at four different distances at three heights, and they were respectively equipped with two wireless modules in each place: the 2.4 GHz band and the 400 MHz band as shown in Figure 7. 

**Figure 8:**
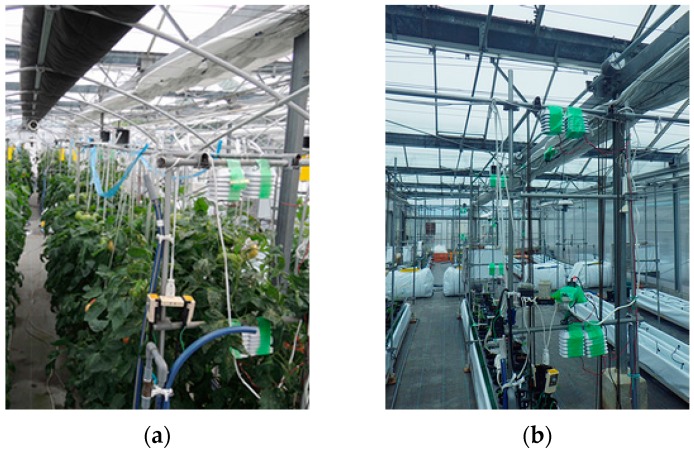
(**a**) Greenhouse environment with lush tomatoes; (**b**) Greenhouse environment without lush tomatoes.

We installed the sensor nodes on four places: the nearest place from the sink node’s antenna (A), the middle of each community (B) and (C) where the scattered light sensor nodes are installed during the operation, and the farthest place from the sink node’s antenna (D).The packet transmission interval was 60 s, and the evaluation period was from 20 March 2015 to 10 June 2015. In this period, there were plants in the greenhouse from 20 March 2015 to 31 May 2015 as shown in Figure 8a, while there were no plants from 1 June 2015 to 10 June 2015 as shown in Figure 8b. Sensor nodes are put in radiation shields to avoid failure during pesticide spraying.

The metrics of this experiment uses the packet error rate (PER). The PER is calculated by the following equation:

(1) $$\text{PER}\left[\%\right]=\frac{\text{Number of packet errors}}{\text{Number of received packets}+\text{Number of packet errors}}\times 100$$

The 400 MHz band wireless module has a retransmission function, but the 2.4 GHz band wireless module has no retransmission function. Therefore, for the sake of fairness packet errors include packet retransmission, which means that the packet was not received at once in this experiment.

**Figure 9:**
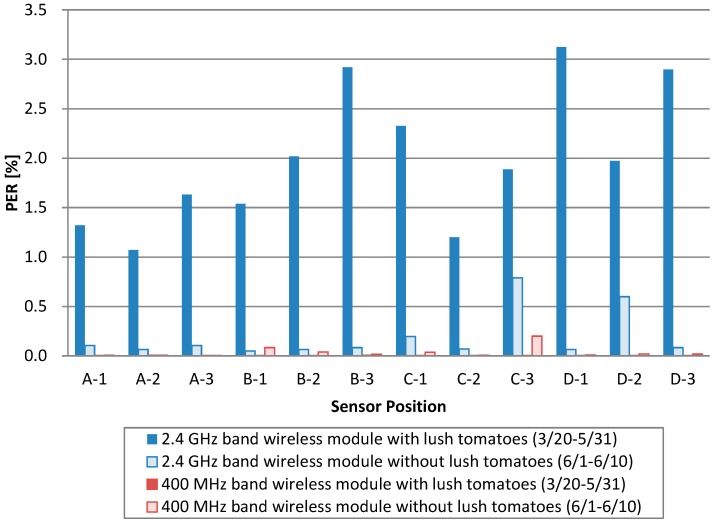
PER result for each sensor.

Figure 9 shows the wireless module comparison in a basic experiment. The blue bars mean the 2.4 GHz band wireless module, and the red bars mean the 400 MHz band wireless module. Besides, the full bars mean there are plants, and the empty bars mean there are no plants. This graph shows that the 2.4 GHz band wireless module with lush tomatoes is where the most packet loss occurred. Each sensor node sent approximately 11 million packets during the cultivation period, but about 1000 to 3000 packet units are wireless communication errors. This means that the PER is 1.0 to 3.0 percent with the 2.4 GHz band wireless module.

On the other hand, the 400 MHz band wireless module had fewer packets even though there were plants. The number of packets lost was four, even in the sensor node with the most packet errors. Only the B-1 node of the 400 MHz band without plants showed an increased packet error; however, the other nodes had fewer packet errors than when using the 2.4 GHz band even when considering packet resend. The PER of the 400 MHz band with the method of IEEE 802.15.6 standard is 0.2 percent at maximum. From this result, the 400 MHz band with the method of IEEE 802.15.6 standard is suitable in a greenhouse where hydroponics methods are used.

**Figure 10:**
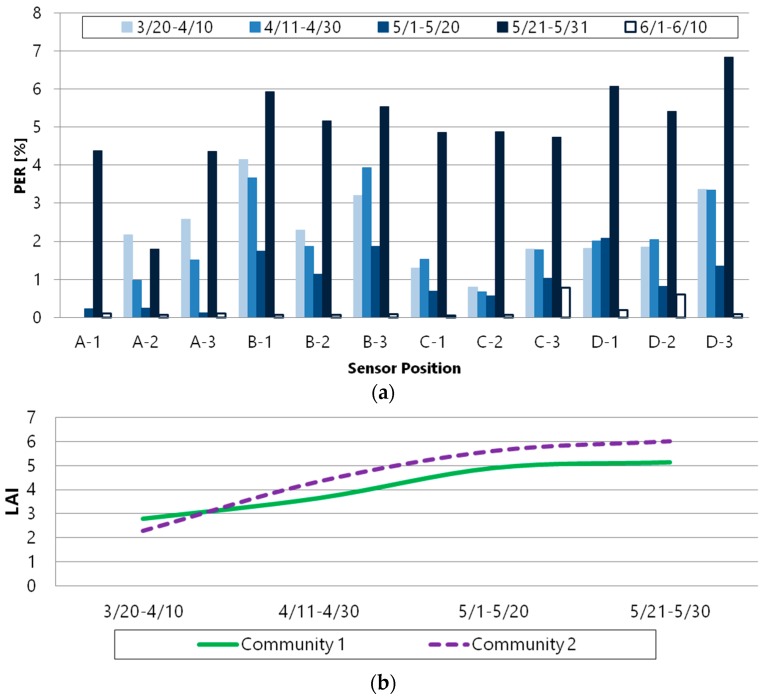
(**a**) PER transition period-to-period; (**b**) LAI transition period-to-period.

We analyzed the packet error at the 2.4 GHz band wireless module in detail. Figure 10a shows the PER, and Figure 10b shows the leaf area index (LAI) transition period-to-period for each module. The LAI is an index indicating the degree to which plants grow leaves and can be indirectly estimated based on RLI because the LAI is strongly inversely proportional to the RLI. Therefore, the lusher the leaves of the plants are the larger the LAI is, and then the line of sight (LoS) is not ensured. In the PER graph in Figure 10a, for all sensor nodes except A-2, the most packet loss occurred from 21 May 2015 to 31 May 2015 when the PER is over 4 percent. Looking at this period, the LAI has not changed compared to the previous period. On the other hand, the PER increased or decreased during the period of changing LAI (20 March 2015 to 21 May 2015).

It is assumed that the environment changes in the path of moving leaves, and the link quality of wireless communication is also changed when the LAI increases. When the leaves move due to growth, it is considered that the radio wave absorption or the radio wave reflection changes. On the other hand, when the LAI is not changed because plant growth was completed, the environment does not change due to the effect of the leaves. Since the period of 21 May 2015 to 31 May 2015 has the highest LAI and the LoS is not ensured in the path, it is thought that this is the reason for the occurrence of the largest number of packet errors.

**Figure 11:**
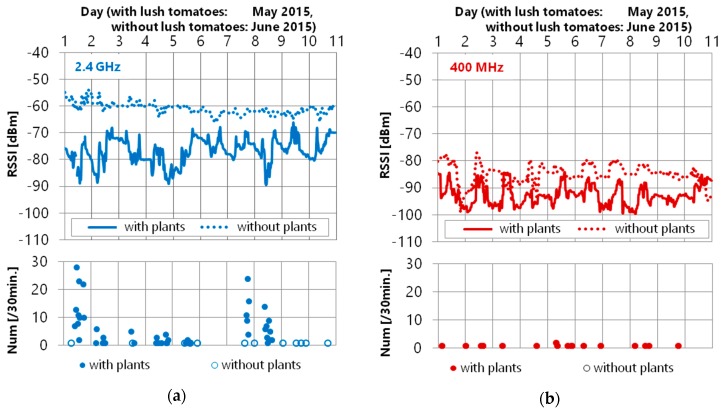
RSSI variation and packet loss with or without plants: (**a**) 2.4 GHz band; (**b**) 400 MHz band.

In addition, we had a detailed analysis of the received packets from sensor nodes and packet errors at sensor position D-3 where the most packet errors occurred from the received signal strength indicator (RSSI) data. Figure 11a is the 2.4 GHz band wireless module, and Figure 11b is the 400 MHz one. The RSSI is lower in the 400 MHz band when comparing the two wireless modules, which is the difference in antenna gain. Therefore, in this section, we compared whether there were plants in each wireless module.

The average RSSI of 2.4 GHz increased after the plants were removed. Besides, the change in width of the RSSI in the 2.4 GHz band wireless module is large when there are plants. In particular, a lot of packet errors occurred when the RSSI fell to −90 dBm. Since such a RSSI decline is not seen when there were no plants, this result means that the 2.4 GHz band wireless module was affected by the plants.

However, the RSSI of the 400 MHz band wireless module did not change much irrespective of whether there were any plants. The packet error (retransmission) in this period is twice per 30 min at most, despite this node being the farthest node from the sink node. Accordingly, it was found that the 400 MHz band is suitable in greenhouse environments with many plants.

In Figure 11b, it can be seen that variation in RSSI occurred with or without plants. This is considered to give more stable wireless communication to investigate the cause of the RSSI variation, which could be the weather, daily work tasks, and so on. Regarding the antenna of 2.4 GHz wireless modules, the antenna is deployed on the outside of the wireless module shown in Figure 4b. On the other hand, the antenna of 429 MHz wireless modules is a chip antenna located on the wireless module. Therefore, we considered that the antenna gain of our 2.4 GHz wireless module is higher than that of the 429 MHz wireless module. We think our experimental results show the usefulness of 429 MHz wireless communication because 429 MHz wireless communications have higher diffraction performance despite their lower antenna gain.

### 3.2. System Evaluation

**Figure 12:**
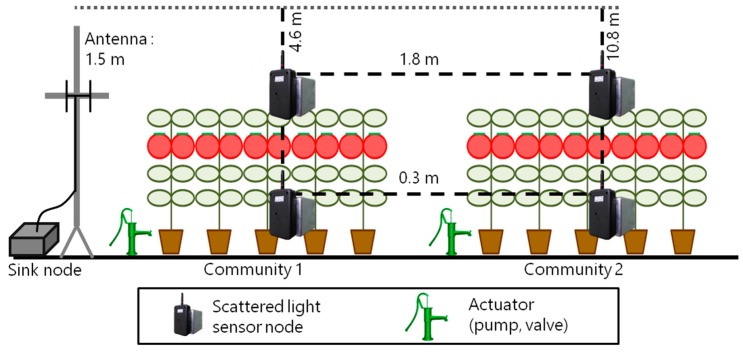
Sensor position for system evaluation.

As confirmed by the effect of the 400 MHz band with the IEEE 802.15.6 standard method, we have conducted an evaluation experiment on the tomato hydroponics system. A set of scattered light sensor nodes was placed in each plant community as shown in Figure 12. We run this system during the tomato cultivation period of about 3 months.

The results of data losses and packet resends for each scattered light sensor node are given in Table 2. 

**Figure 13:**
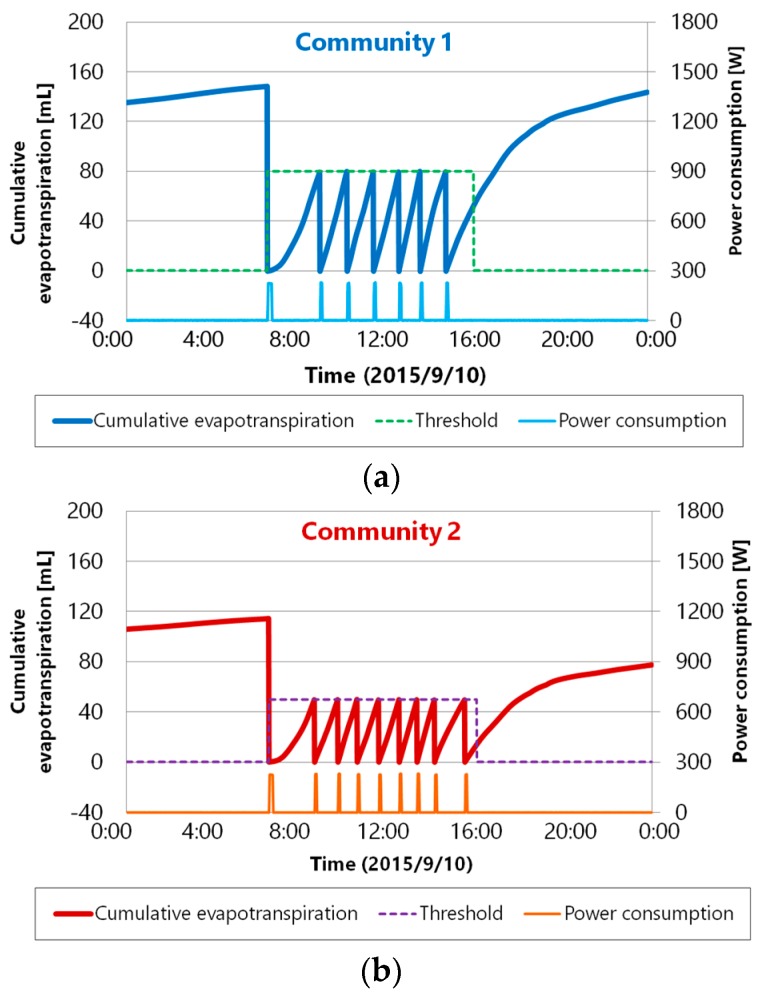
Result of hydroponic liquid control (**a**) community 1; (**b**) community 2.

Except for with the community 1 upper sensor node, some packet resends occurred, but all of the sensor nodes were able to run without data loss. Therefore, although some retransmission was necessary, we achieved reliable data collection without data loss. Figure 13a,b show the hydroponic liquid control records of each community. Supply control is performed according to the control settings in Table 3. There is also a sensor for measuring the power consumption at the pump supplying the hydroponic liquid, and we were thus able to confirm whether the pump was on or off. 

Figure 13 shows that the pump runs immediately when the cumulative evapotranspiration reaches the threshold value in the control period. Due to the successful realization of wireless communication without data loss, we achieved precise control for tomato hydroponics on a minute scale, which is essential for high-quality tomato cultivation.

## 4. Conclusions

In this paper, we proposed a highly reliable wireless control system for tomato hydroponics. To address the challenge of using wireless sensor networks in an agricultural field, we adopted the 400 MHz band and the IEEE 802.15.6 standard method for a highly reliable WSN. Besides, we performed software implementation of a selection of fault-tolerant or interoperable hardware in a high temperature and high humidity environment. Then we installed the tomato hydroponics control system and operated it in a greenhouse. The experiments show the application effect of the 400 MHz band and the method of IEEE 802.15.6 standard as follows:

- The 400 MHz band is less affected by plants compared to the 2.4 GHz band.
- Although there are some packet retransmissions, the 400 MHz band and the IEEE 802.15.6 standard could reduce packet errors and data losses.
- The scattered light sensor node with 400 MHz band could control hydroponics on a minute scale.

In our current prototype system, we focused on whether a reliable WSN could be built using the 429 MHz band wireless communications based on the IEEE 802.15.6 standard. If we could use 2.4 GHz wireless modules implemented based on the same standard as the 400 MHz wireless modules, we could evaluate the effect due to the frequency difference. In future work, we would like to evaluate the effect due to the difference in the frequency in more detail and the power saving performance of our developed reliable wireless control system. Moreover, a remaining issue is evaluation of the effect of increasing the number of sensor nodes up to 40 and verification whether the whole farm can be covered by one sink node. Besides, we have to show our developed system is useful for hydroponics methods under other conditions and in other environments.

## Figures and Tables

**Table 1:** Specifications of the wireless module.

Specification	2.4 GHz Band Wireless Module	400 MHz Band Wireless Module
Chip	CC2420 (Texas Instruments)	CC430F5137 (Texas Instruments)
Frequency (Bandwidth)	2405–2480 MHz (5 MHz interval 16ch)	429.25–429.75 MHz (12.5 kHz interval 40ch)
Protocol	Based on IEEE 802.15.4 standard	Based on IEEE 802.15.6 standard
Bit rate	250 kbps	7200 bps
Transmit power	10 mW	10 mW
Power supply	3-volt DC	3-volt DC

**Table 2:** Data losses and packet resends of system prototype.

Sensor Placement	Receive/Send ^1^	Data Losses	Packet Resends
Community 1 Upper	86475 / 86475	0	0
Community 1 Inner	87426 / 87453	0	27
Community 2 Upper	84991 / 85110	0	119
Community 2 Inner	89687 / 89702	0	15

^1^ The difference in the number of sent packets is caused by artificial factors, for example, battery replacement.

**Table 3:** Control settings of each tomato community.

Placement	Control Period	Threshold	Supply Time
Community 1	7 am–5 pm	80	5
Community 2	7 am–5 pm	50	5
